# Neurogenic Thoracic Outlet Syndrome Caused by Vascular Compression of the Brachial Plexus: A Report of Two Cases

**DOI:** 10.1055/s-0037-1607977

**Published:** 2018-02-28

**Authors:** Amgad Hanna, Larry O'Neil Bodden, Gabriel R. L. Siebiger

**Affiliations:** 1 Department of Neurological Surgery, University of Wisconsin, Madison, Wisconsin, United States; 2 Universidade Federal de Ciências da Saúde de Porto Alegre, Porto Alegre, Brazil

**Keywords:** thoracic outlet syndrome, vascular compression, microvascular decompression

## Abstract

Thoracic outlet syndrome (TOS) is caused by compression of the brachial plexus and/or subclavian vessels as they pass through the cervicothoracobrachial region, exiting the chest. There are three main types of TOS: neurogenic TOS, arterial TOS, and venous TOS. Neurogenic TOS accounts for approximately 95% of all cases, and it is usually caused by physical trauma (posttraumatic etiology), chronic repetitive motion (functional etiology), or bone or muscle anomalies (congenital etiology). We present two cases in which neurogenic TOS was elicited by vascular compression of the inferior portion of the brachial plexus.

## Introduction

Thoracic outlet syndrome (TOS) is caused by compression of the brachial plexus and/or subclavian vessels as they pass through the cervicothoracobrachial region, exiting the thoracic girdle. 1 There are three main types of TOS: neurogenic TOS, arterial TOS, and venous TOS. Neurogenic TOS accounts for approximately 95% of all cases, and it is usually caused by physical trauma (posttraumatic etiology), chronic repetitive motion (functional etiology), or bone or muscle anomalies (congenital etiology). 2 We present two cases in which neurogenic TOS was elicited by vascular compression of the inferior portion of the brachial plexus.

## Case 1

A 39-year-old left-handed woman presented to the neurosurgery clinic with a history of right-sided arm and shoulder numbness for almost 5 years. Her occupation involved desk work. She had been previously diagnosed with neurogenic TOS due to the presence of numbness in her right shoulder and arm radiating down the hand and fingers in association with right neck tightness. She underwent physical therapy that included therapeutic exercise, soft-tissue and joint mobilization, neuromuscular reeducation, and taping, but these did not alleviate her symptoms. Specific discomfort affected the right shoulder both anteriorly and posteriorly, radiating down the arm, occasionally affecting either the medial two digits or the lateral three digits, with sporadic numbness in all digits. There were no symptoms in the left arm, except very mild infrequent numbness. Symptoms did not change according to the weather, tended to worsen while sleeping or with overhead movements, and improved with physical activities in general, except running. She had a past medical history of migraine.

On physical examination, motor examination showed 5/5 strength in all muscle groups. Deep tendon reflexes revealed biceps reflex spreading bilaterally, otherwise normal reflexes were elicited in both upper and lower extremities. She had a right-sided Hoffmann's sign. All thoracic outlet maneuvers were positive, including Adson's test, Wright's test, and Roos' test, with sensation of cold and numbness with arm elevation on the right side. She had a Tinel's sign over the right infraclavicular brachial plexus and percussion tenderness over the right supraclavicular brachial plexus.

Nerve conduction studies revealed mild right spinal accessory neuropathy, which is considered to be incidental.

CT scan of the cervical spine showed a cervical rib bilaterally at C7. Doppler vascular studies showed bilateral impingement on the subclavian artery with thoracic outlet maneuvers. Magnetic resonance imaging of the cervical spine and of the brachial plexus ruled out both foraminal stenosis and the presence of any masses.

In light of the failure of conservative therapy, the patient was offered a right thoracic outlet decompression with removal of the cervical rib.

**Fig. 1:**
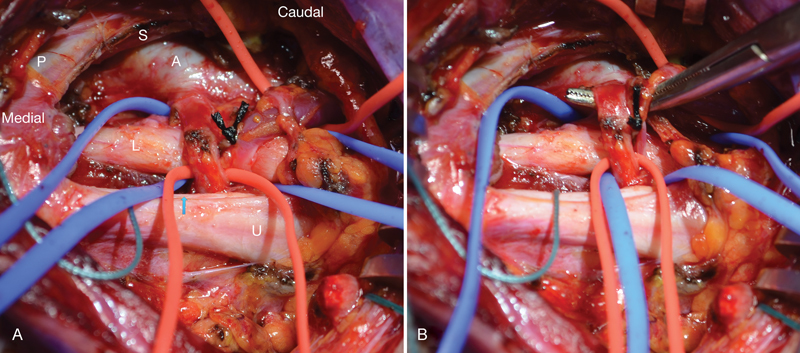
First case. ( **A** ) Right brachial plexus exposure showing compression of the lower trunk of the brachial plexus (L) by a branch from the subclavian artery (A) and a vein. Scalenus anterior muscle edge after partial scalenectomy (S), phrenic nerve (P), upper trunk (U), middle trunk (blue arrow). ( **B** ) A right-angled instrument is used to lift up the vessels off the lower trunk of the brachial plexus. Shredded Teflon is then used for separation.

Surgery was performed through a standard right supraclavicular approach. The anterior scalene muscle was resected including the fascial band underneath. The C7 rib was identified and resected with Kerrison rongeurs. The dorsal scapular artery was noticed coming off of the subclavian artery, and ran directly over the inferior portion of the brachial plexus, clearly compressing it. It was thought that pulsations from this artery could add to the compressive pathology on the nerve, and shredded non-adherent cotton was slipped underneath the artery to separate it from the nerve ( Fig. 1 ). Upon detailed examination, the artery felt widely decompressed and free, and vascular decompression was thought to have been achieved at this point. Closure was performed per routine.

At 3-month follow-up, the patient reported a 70% improvement in numbness. At 1 year, her numbness was almost completely gone, and electromyography performed 2 years after the surgical procedure was normal. At 30-month follow-up, the patient had complete resolution of pain.

## Case 2

**Fig. 2:**
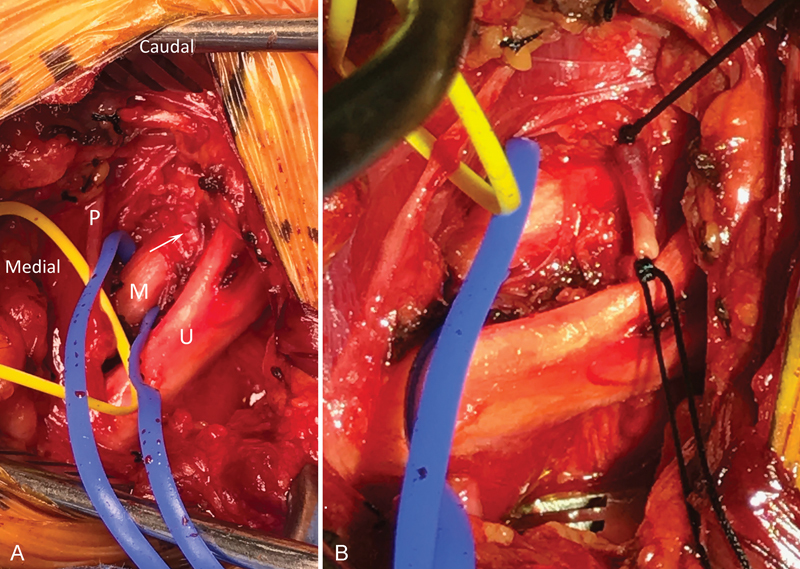
Second case. ( **A** ) A vessel (arrow) was found to pass between the middle (M) and upper (U) trunks of the brachial plexus. This was ligated and cut ( **B** ). P, phrenic nerve.

A 46-year-old female presented with pain and numbness in both upper extremities radiating to the medial two fingers. Thoracic outlet maneuvers were positive bilaterally. She had a partial response to botulinum toxin injection to the anterior scalene. At surgery, a branch of the subclavian artery was riding over the middle trunk of the brachial plexus compressing it before it dove posteriorly between the middle and upper trunks. This branch was ligated and cut ( Fig. 2 ). Postoperatively, her symptoms significantly improved. At 6 weeks, she reported 75% improvement in her preoperative pain.

## Comment

In these two case reports, we describe an unusual cause of neurogenic TOS due to vascular compression of the lower brachial plexus by a branch from the subclavian artery, likely the dorsal scapular artery. Recent literature has begun to describe this pathology both in cadaveric studies and surgical cases. 3 4 5 The first case is unusual in the use of cushioning instead of ligation in the treatment of the vascular compression. The technique is adopted from what has been used for microvascular decompression of the trigeminal nerve. In regards to the overall presentation, TOS is still often considered a diagnosis of exclusion with neurogenic TOS, the most challenging variant to diagnose with several clinical maneuvers being developed in recent years. 6 Initial treatment is rehabilitation physiotherapy, but patients who fail the conservative treatment are known to frequently benefit from surgical decompression. 7 Our patient followed current standard of practice, initially with physical therapy and, in the absence of improvement, was offered surgery. She ultimately achieved significant relief of her symptoms. Special attention should be given by surgeons during the procedures to identify and treat vascular compressive anatomy that may lead to neurogenic TOS.
